# Healthcare professionals’ promotion of physical activity to people living with and beyond head and neck cancer: a cross-sectional survey

**DOI:** 10.1007/s00520-024-09027-8

**Published:** 2024-12-03

**Authors:** Hannah C. Doughty, Kerry Woolfall, Ruaraidh A. Hill, Adrian W. Midgley, Joanne M. Patterson, Lynne M. Boddy, Simon N. Rogers, Nefyn H. Williams

**Affiliations:** 1 Department of Primary Care and Mental Health, University of Liverpool, Liverpool, L69 3GL UK; 2 Faculty of Science, Liverpool John Moores University, Liverpool, L3 3AF UK; 3 Department of Public Health, Policy and Systems, University of Liverpool, Liverpool, L69 3GL UK; 4 Liverpool Reviews and Implementation Group, Department of Health Data Science, University of Liverpool, Liverpool, L69 3GL UK; 5 Department of Sport and Physical Activity, Edge Hill University, Ormskirk, L39 4QP UK; 6 Liverpool Head and Neck Centre, University of Liverpool, Liverpool, L69 3GB UK; 7 The Physical Activity Exchange, Research Institute for Sport and Exercise Sciences, Liverpool John Moores University, Liverpool, L3 2EX UK; 8 Head and Neck Centre, Wirral University Teaching Hospital, Wirral, CH49 5PE UK

**Keywords:** Barrier, Behavior change, Cancer, Exercise, Facilitator, Guideline

## Abstract

**Purpose:**

Physical activity (PA) can improve health-related outcomes for head and neck cancer (HaNC) patients, and PA guidance from healthcare professionals’ can increase patients’ PA levels. However, less than 9% of HaNC patients are physically active. This study explored healthcare professionals’ promotion of PA across the National Health Service (NHS) in North West England and North Wales, to HaNC patients.

**Methods:**

A cross-sectional online survey exploring healthcare professionals’ promotion of PA in HaNC. The International Physical Activity Questionnaire–Short Form was used to estimate healthcare professionals’ PA levels. Quantitative data were analyzed using descriptive or inferential statistics and qualitative data were analyzed using reflexive thematic analysis. Data were synthesized drawing on the capability-opportunity-motivation-behavior model and theoretical domains framework.

**Results:**

Eighty-one professionals participated. Fifty-three percent self-reported high levels of PA. Seventy-five percent considered PA promotion as part of their role; however, only 39% discussed PA with their patients *(reflective motivation and social/professional role and identity)*. Only 38% felt confident initiating PA discussions and 76% reported needing further training. Training on the benefits of PA for HaNC patients and how to encourage health-related behavior change were identified as beneficial *(psychological capability and knowledge)*.

**Conclusion:**

Healthcare professionals are influential in enabling patients to adopt health-related behavior change; however, PA promotion was not routine practice for professionals involved in the care of HaNC patients. Training should be provided to professionals on PA promotion, with a focus on behavior change techniques. Future research should explore how behavior change techniques can be implemented into clinical practice to improve health-related outcomes in HaNC.

**Supplementary Information:**

The online version contains supplementary material available at 10.1007/s00520-024-09027-8.

## Introduction

Incidence rates for head and neck cancer (HaNC) are increasing, and there are approximately 12,400 new cases in the United Kingdom (UK) every year [1]. HaNC treatment is complex, and patients can experience challenging treatment-related side effects including dyspnea, fatigue, social anxiety, and isolation [2–6]. Physical activity (PA) can decrease fatigue and improve functional and psychological well-being for HaNC patients [7–9]. The percentage of patients who are physically active is significantly lower in HaNC compared with other cancers [10–12]. Thirty percent of people with breast cancer, and 25% of people with colorectal cancer were reported to meet UK recommended PA levels of at least 75 min of vigorous-intensity PA, 150 min of moderate-intensity PA, or an equivalent combination per week, and muscle-strengthening PA on two or more days a week [10, 11]. However, only 9% of HaNC patients were reported to meet these recommendations [12]. PA recommendations from healthcare professionals can increase patients’ PA levels [13] and Macmillan Cancer Support have outlined that PA should be incorporated as part of standard National Health Service (NHS) cancer care [14]. However, fewer than half of UK cancer specialists routinely discuss PA with their patients [15]. Healthcare professionals have reported lack of time, knowledge, and training in PA promotion, as barriers to their promotion of PA [16, 17], and healthcare professionals’ own PA levels can influence their PA promotion [18]. Behavior change theory is useful to understand PA promotion and participation and to develop strategies that can be applied to practice. Cross-sectional studies have explored healthcare professionals’ PA promotion to people with cancer [15, 17–29]; however, none have explored whether PA is promoted across the NHS in the UK, to HaNC patients. The primary aim of the present study was to explore healthcare professionals’ promotion of PA to HaNC patients, using the capability-opportunity-motivation-behavior (COM-B) model [30] and the theoretical domains framework (TDF) [31]. Secondary aims were to (1) explore if there were any associations between healthcare professionals’ own levels of PA and their PA promotion and (2) explore if there were any associations between a healthcare professional’s role and their promotion of PA.

## Methods

### Study design

A cross-sectional online survey of healthcare professionals working in HaNC.

### Participant recruitment

This research was conducted across the North West of England and North Wales between August 2021 and January 2022. Healthcare professionals were recruited using a combination of voluntary and snowball sampling techniques [32, 33], including NHS participation identification centers (PIC), and online advertisements. Individuals who were recruited through PICs were provided with study information by a member of the clinical team. Individuals recruited through snowball sampling or online methods were provided with study information, or directly contacted the research team. Healthcare professionals were eligible if they were involved in the care of HaNC patients and practising in North West England or North Wales. Ethical approval was granted by the Greater Manchester West NHS Research Ethics Committee (REC) (REC: 21/NW/0108; IRAS ID: 293302), and informed consent was obtained from all participants.

## Materials

### International Physical Activity Questionnaire–Short Form (IPAQ-SF)

The self-administrated IPAQ-SF [34] was used to assess healthcare professionals’ self-reported levels of PA. The IPAQ is a validated measure of self-reported PA among individuals aged 18–69 years and consists of seven questions relating to moderate-vigorous-intensity activity, walking, and sitting behavior. PA levels were assessed using metabolic equivalent of task minutes per week (MET-min/week), and MET-min/week scores were calculated using the IPAQ-SF scoring protocol [35]. The total of vigorous, moderate, and walking activities created a total MET-min/week PA score. These scores were used to categorize individuals into one of the following categories: category one: low levels of PA; category two: moderate levels of PA; and category three: high levels of PA. The IPAQ-SF produced repeatable data (Spearman’s *p* clustered around 0.8) and criterion validity had a median *p* of approximately 0.30 [34].

### Study procedure

This survey was based on a previous survey that assessed general practitioners’ knowledge, use, and confidence in applying national PA guidelines and assessment tools in England [36]. Questions were developed and adapted to fit the purpose of the present study. Permission to use the survey was obtained from the corresponding author. The survey was piloted with a small number of the target population (see Online Resource 1). Five questions were removed and the final survey consisted of 29 items which included open and close-ended questions (see Table 3). Optional open-text boxes were provided under each survey question. The participant information sheet, consent form, and online survey were made available through the web-based survey tool Qualtrics (Qualtrics, Provo, UT). Participants were allocated with an anonymized unique identification number.

### Data analysis

**Fig. 1:**
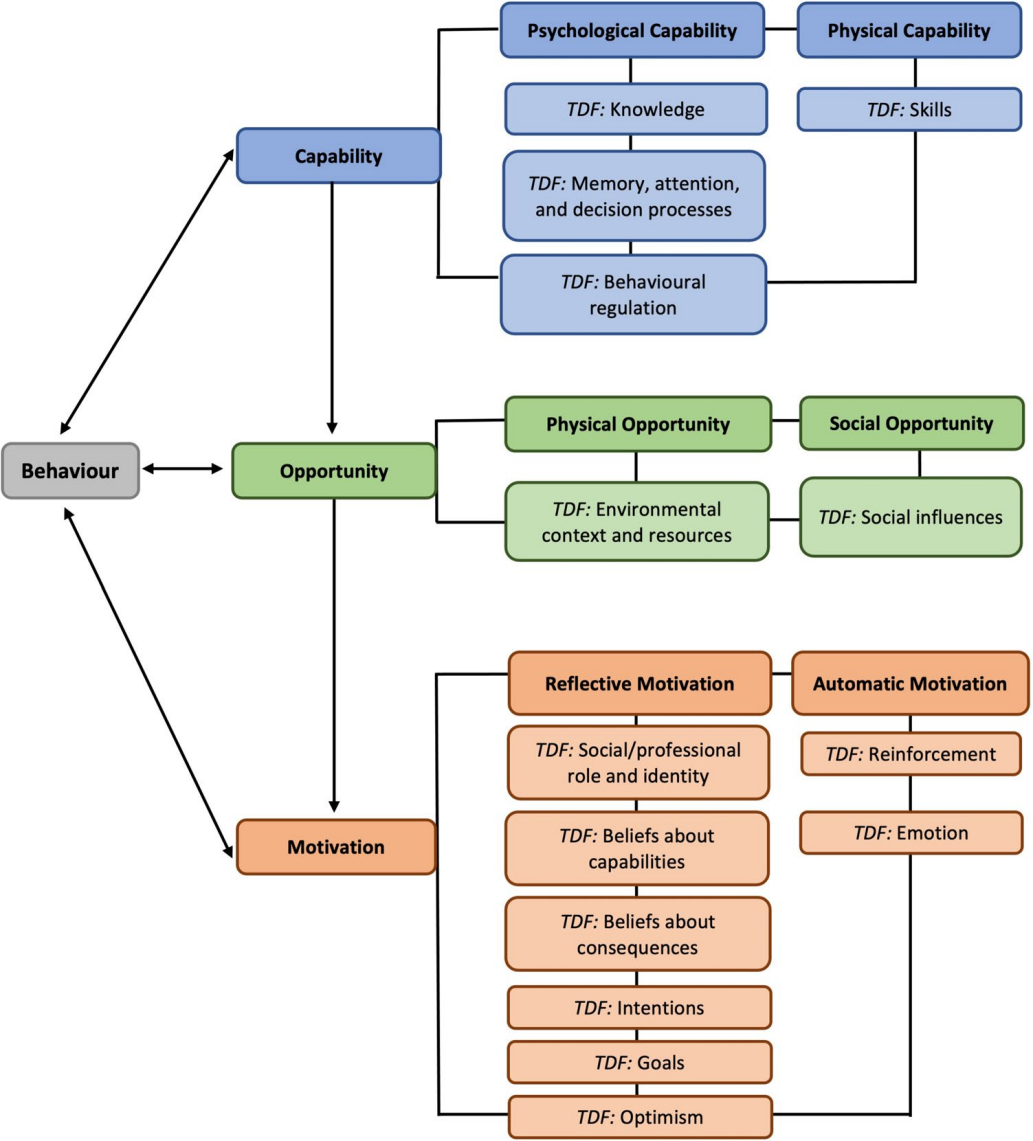
Capability-opportunity-motivation-behavior (COM-B) model [30] and theoretical domains framework (TDF) [31] behavior change domains (adapted from [40])

Data were imported from Qualtrics into Microsoft Excel (Microsoft Corporation, USA). IPAQ-SF data were analyzed according to their scoring protocols and healthcare professionals were categorized into whether they met the Chief Medical Officers’ (CMO) PA guidelines for adults, relating to the amount of aerobic PA conducted per week [37]. Quantitative data were analyzed using descriptive or inferential statistics in IBM SPSS Statistics for MacOS, version 28 (SPSS Inc., IBM, Chicago, IL). Percentages were rounded to the nearest integer and may not equal 100%. Qualitative data were organized using NVivo 12 for MacOS (released in 2018; QSR International Pty Ltd., Burlington, MA, USA) and Microsoft Word. Qualitative data were analyzed using reflexive thematic analysis [38]. Data were synthesized using constant comparison [39] and deductively mapped to the relevant COM-B constructs [30] and TDF domains [31]. The COM-B model posits that behavior change is dependent upon an individual possessing the capability, opportunity, and motivation in order to change their behavior [30]. The TDF builds on the COM-B and consists of 14 domains that further understand the underlying barriers to, and facilitators of, behavior change [31] (see Fig. 1).

### Inferential analyses

Exploratory analyses were conducted to assess whether there were associations between (1) a professional’s role and their promotion of PA and (2) a professional’s own PA levels and their promotion of PA (see Table 1). IBM SPSS Statistics was used for all statistical analyses and total MET-min/week PA values were used in the IPAQ-SF inferential analyses. Normality of observed data were assessed in IBM SPSS using standard graphical methods (box and whisker plots and quantile–quantile plots) and data were not normally distributed. Professional roles were coded into four categories (allied health, nursing, medical, and managerial). The managerial category was removed from the analyses as only one manager completed the survey. Chi-square tests of independence (*χ*^2^) compared groups of categorical data [41], and Mann–Whitney *U*-tests [41] or Kruskal–Wallis *H*-tests [41] compared groups of ordinal and continuous data. Effect sizes were calculated by hand using standard methods for Mann–Whitney *U*-tests and Kruskal–Wallis *H*-tests [42], and using IBM SPSS for Chi-square tests of independence. The strength of effect sizes are presented in Table 2.

**Table 1:** Variables used in the inferential analyses

Independent variables	Dependent variables
• Healthcare professional role • Total MET-min/week PA levels	• Whether professionals considered it as part of their role to discuss PA with their patients • Whether professionals had any concerns having these discussions • Professionals knowledge of where to signpost patients for further information about PA • Professionals confidence initiating PA discussions • Professionals frequency of PA discussions • Whether professionals required further training to feel confident initiating PA discussions

**Table 2:** Strength of effect sizes for statistical tests [43]

Chi-square tests of independence (*v*)	Kruskal–Wallis *H*-tests (*η*^*2*^*)*	Mann–Whitney *U*-tests (*r*)
• 0.1 was considered a ‘small’ effect • 0.3 was considered a ‘medium’ effect • 0.5 was considered a ‘large’ effect	• 0.01 was considered a ‘small’ effect • 0.06 was considered a ‘medium’ effect • 0.14 was considered a ‘large’ effect	• 0.1 was considered a ‘small’ effect • 0.3 was considered a ‘medium’ effect • 0.5 was considered a ‘large’ effect

## Results

### Participants

A total of 81 healthcare professionals participated; 93% fully completed the survey and 7% partially completed the survey. Participants were mainly practising in the North West of England (95%) and worked across a variety of healthcare settings (Table 3).

**Table 3:** Survey responses (*N* = 81)

Survey item	*N* (%)
1. What gender do you identify as?	
Female	59 (73%)
Male	20 (25%)
Non-binary	1 (1%)
Data not reported	1 (1%)
2. What is your sexuality?	
Heterosexual	70 (86%)
Homosexual	5 (6%)
Bisexual	1 (1%)
Other	2 (2%)
Data not reported	3 (4%)
3. What is your ethnicity?	
White—English/ Irish/ Gypsy or Irish traveller	68 (84%)
Asian/Asian British	7 (9%)
Other ethnic group	3 (4%)
Data not reported	3 (4%)
4. What is your age?	
Range	23 – 57 years
Mean	40
Standard deviation	10
Data not reported	4 (5%)
5. Approximately how long have you worked as a healthcare professional?	
Range	8 months – 38 years
Median	14.5
Interquartile range	18
Data not reported	1 (1%)
6. Approximately how long have you worked with head and neck cancer patients?	
Range	1 – 33 years
Median	7.5
Interquartile range	12
Data not reported	1 (1%)
7. What is your current job role?	
Radiography *(Allied Health)*	19 (23%)
Nursing *(Medical)*	19 (23%)
Speech and language therapy *(Allied Health)*	12 (15%)
Dietetics *(Allied Health)*	5 (6%)
General practice *(Medical)*	5 (6%)
Consultant [not defined] *(Medical)*	4 (5%)
Oral and maxillofacial surgery *(Medical)*	4 (5%)
Healthcare *(Allied Health)*	3 (4%)
Physiotherapy *(Allied Health)*	2 (2%)
Clinical education *(Allied Health)*	2 (2%)
Managerial	2 (2%)
Radiology *(Medical)*	1 (1%)
Dentistry *(Medical)*	1 (1%)
Data not reported	2 (2%)
8. Where is your location of practice?	
North West of England	77 (95%)
North Wales	2 (2%)
Both	1 (1%)
Data not reported	1 (1%)
9. Approximately how many head and neck patients do you see a month?	
Range	1 – 300 patients
Median	20
Interquartile range	36
Data not reported	15 (19%)
10. What is the name of the organization you work in?	
Liverpool University Hospitals NHS Foundation Trust	27 (33%)
The Christie NHS Foundation Trust	13 (16%)
The Clatterbridge Cancer Centre	8 (10%)
Lancashire Teaching Hospitals NHS Foundation Trust	6 (7%)
Southport and Ormskirk Hospital NHS Trust	3 (4%)
East Lancashire Hospitals NHS Trust	3 (4%)
Mersey Care NHS Foundation Trust	2 (2%)
Mid Cheshire Hospitals NHS Foundation Trust	2 (2%)
St Georges Medical Centre (GP)	3 (4%)
Betsi Cadwaladr University Health Board	1 (1%)
Lakeside Physiotherapy	1 (1%)
Marine Lake Medical Practice (GP)	1 (1%)
Countess of Chester Hospital	1 (1%)
St. Marks Dee View Surgery (GP) and Arrowe Park Hospital	1 (1%)
Warrington and Halton Hospitals NHS Trust	1 (1%)
Data not reported	8 (10%)
11. Do you think that it is within the remit of your role to discuss physical activity with people living with and beyond head and neck cancer?	
Yes	61 (75%)
No	19 (23%)
Data not reported	1 (1%)
12. Do you have any concerns discussing physical activity with head and neck cancer patients, or signposting patients to existing physical activity provision in the area?	
Yes	34 (42%)
No	47 (58%)
13. When do you think would be an appropriate time to discuss physical activity with head and neck cancer patients?*	
At one time-point	15 (19%)
At all time-points (At diagnosis, pre-treatment, during-treatment, and post-treatment)	62 (77%)
Other	3 (4%)
Data not reported	1 (1%)
14. What in your opinion do you think are the barriers that head and neck cancer patients may face when being physically active?	
Data reported	79 (98%)
Data not reported	2 (2%)
15. In your opinion, what do you think could be done to overcome these barriers?	
Data reported	71 (88%)
Data not reported	10 (12%)
16. A) Are you familiar with the following guidelines or exercise referral schemes? B) If familiar, do you use the guideline(s) or exercise referral schemes?**	
A) Chief Medical Officers’ (CMO) Physical Activity Guidelines?	
Not at all familiar	39 (48%)
Slightly familiar	16 (20%)
Somewhat familiar	11 (14%)
Moderately familiar	14 (17%)
Extremely familiar	1 (1%)
B) I use these guidelines**	
Strongly disagree	1 (2%)
Disagree	10 (24%)
Neither agree nor disagree	14 (33%)
Agree	16 (38%)
Data not reported	1 (2%)
A) Are you familiar with any of the National Institute for Health and Care Excellence (NICE) physical activity guidelines?	
Not at all familiar	33 (41%)
Slightly familiar	18 (22%)
Somewhat familiar	20 (25%)
Moderately familiar	9 (11%)
Extremely familiar	1 (1%)
B) Which guideline(s) are you aware of AND do you use them?**	
Data reported	15 (31%)
Data not reported	33 (69%)
A) Are you familiar with the Macmillan guidelines for promoting physical activity for people living with and beyond cancer?	
Not at all familiar	32 (40%)
Slightly familiar	21 (26%)
Somewhat familiar	13 (16%)
Moderately familiar	8 (10%)
Extremely familiar	5 (6%)
Data not reported	2 (2%)
B) I use these guidelines**	
Strongly disagree	3 (6%)
Disagree	8 (17%)
Neither agree nor disagree	13 (28%)
Agree	16 (34%)
Strongly agree	1 (2%)
Data not reported	6 (13%)
A) Are you familiar with any exercise referral schemes available for people living with and beyond head and neck cancer?	
Not at all familiar	55 (68%)
Slightly familiar	7 (9%)
Somewhat familiar	9 (11%)
Moderately familiar	5 (6%)
Extremely familiar	3 (4%)
Data not reported	2 (2%)
B) Which guideline(s) are you aware of AND do you use them? (open-text)**	
Data reported	13 (54%)
Data not reported	11 (46%)
17. A) Which, if any, of the following physical activity assessment tools are you aware of to help assess patients’ physical activity levels? B) If aware, do you use the tool(s)?**	
A) General Practice Physical Activity Questionnaire (GPPAQ)	
Yes	4 (5%)
No	72 (89%)
Data not reported	5 (6%)
B) If aware, do you use it?**	
Sometimes	1 (25%)
Never	3 (75%)
A) International Physical Activity Questionnaire (IPAQ)	
Yes	4 (5%)
No	77 (95%)
B) If aware, do you use it?**	
Sometimes	1 (25%)
Never	3 (75%)
A) Single-item measure for physical activity	
Yes	4 (5%)
No	77 (95%)
B) If aware, do you use it?**	
Sometimes	2 (50%)
Never	2 (50%)
A) Scottish Physical Activity Screening Questionnaire (Scot-PASQ)	
Yes	1 (1%)
No	80 (99%)
B) If aware, do you use it?**	
Never	1 (100%)
A) English Physical Activity Screening Questionnaire (Eng-PASQ)	
Yes	4 (5%)
No	77 (95%)
B) If aware, do you use it?**	
Sometimes	1 (25%)
Never	3 (75%)
A) Device-based methods for obtaining physical activity data (e.g., Fitbit, pedometer)	
Yes	39 (48%)
No	42 (52%)
B) If aware, do you use it?**	
Frequently	2 (5%)
Sometimes	17 (44%)
Never	20 (51%)
18. I understand how to use these tools in day-to-day practice	
Strongly disagree	29 (36%)
Disagree	21 (26%)
Neither agree nor disagree	13 (16%)
Agree	7 (9%)
Strongly agree	5 (6%)
Data not reported	6 (7%)
19. I discuss physical activity with all my patients	
Strongly disagree	8 (10%)
Disagree	31 (38%)
Neither agree nor disagree	11 (14%)
Agree	20 (25%)
Strongly agree	5 (6%)
Data not reported	6 (7%)
20. I rarely discuss physical activity with all my patients	
Strongly disagree	7 (9%)
Disagree	32 (40%)
Neither agree nor disagree	12 (15%)
Agree	16 (20%)
Strongly agree	8 (10%)
Data not reported	6 (7%)
21. Do you know where to signpost head and neck cancer patients for further information about physical activity?	
Yes	31 (38%)
No	43 (53%)
Data not reported	7 (9%)
22. I am confident initiating physical activity discussions with head and neck cancer patients	
Strongly disagree	7 (9%)
Disagree	18 (22%)
Neither agree nor disagree	19 (23%)
Agree	23 (28%)
Strongly agree	8 (10%)
Data not reported	6 (7%)
23. I feel that I need further training in order to feel confident initiating discussions about physical activity with head and neck cancer patients	
Strongly disagree	2 (2%)
Disagree	6 (7%)
Neither agree nor disagree	6 (7%)
Agree	27 (33%)
Strongly agree	35 (43%)
Data not reported	5 (6%)
24. What training do you think you or your clinical practice would benefit from? (open-text)**	
Data reported	6 (10%)
Data not reported	56 (90%)
25. A) Which, if any, of the following training sessions have you undertaken with respect to encouraging physical activity? B) If you have received training, do you feel more confident?**	
A) Using General Practice Physical Activity Questionnaire (GPPAQ) in practice	
Yes	1 (1%)
No	74 (91%)
Data not reported	6 (7%)
B) If you have received training, do you feel more confident?**	
Yes	1 (100%)
A) Delivering brief interventions to encourage patient’s physical activity	
Yes	14 (17%)
No	61 (75%)
Data not reported	6 (7%)
B) If you have received training, do you feel more confident?**	
Yes	8 (57%)
Somewhat	5 (36%)
Data not reported	1 (7%)
A) Motivational interviewing	
Yes	15 (19%)
No	60 (74%)
Data not reported	6 (7%)
B) If you have received training, do you feel more confident?**	
Yes	5 (33%)
Somewhat	7 (47%)
No	1 (7%)
Data not reported	2 (13%)
A) Use of physical activity assessment tools	
Yes	2 (2%)
No	72 (89%)
Data not reported	7 (9%)
B) If you have received training, do you feel more confident?**	
Yes	1 (50%)
No	1 (50%)
A) Clinical Commissioning Group (CCG) training session on physical activity	
Yes	1 (1%)
No	74 (91%)
Data not reported	6 (7%)
B) If you have received training, do you feel more confident?**	
Data not reported	1 (100%)
A) In-practice training session on physical activity	
Yes	1 (1%)
No	74 (91%)
Data not reported	6 (7%)
B) If you have received training, do you feel more confident?**	
Yes	1 (100%)
A) Royal College of General Practitioners (RCGP) accredited Continuing Medical Education (CME) module on physical activity	
No	75 (93%)
Data not reported	6 (7%)
A) British Medical Journal (BMJ) Physical Activity Module	
No	75 (93%)
Data not reported	6 (7%)
A) Physical Activity Clinical Champions Programme	
Yes	1 (1%)
No	74 (91%)
Data not reported	6 (7%)
B) If you have received training, do you feel more confident?**	
No	1 (100%)
26. The discussions I have about physical activity with my head and neck cancer patients have changed since COVID-19	
Strongly disagree	7 (9%)
Disagree	18 (22%)
Neither agree nor disagree	30 (37%)
Agree	10 (12%)
Strongly agree	7 (9%)
Data not reported	9 (11%)
27. Can you briefly summarise what has changed? (open-text)**	
Data reported	14 (82%)
Data not reported	3 (18%)
28. How frequently do your head and neck cancer patients initiate discussions about physical activity with you?	
Never	12 (15%)
Very rarely	25 (31%)
Rarely	19 (23%)
Occasionally	16 (20%)
Very frequently	1 (1%)
Data not reported	8 (10%)
29. Approximately how frequently have you discussed physical activity with head and neck cancer patients in the last month?	
Never	14 (17%)
Very rarely	15 (19%)
Rarely	12 (15%)
Occasionally	19 (23%)
Very frequently	9 (11%)
Always	4 (5%)
Data not reported	8 (10%)
IPAQ-SF Questionnaire	
Data reported	75 (93%)
Data not reported	6 (7%)

^*^Multiple response options could be selected

^**^Question contingent on the previous response

### IPAQ-SF data

Fifty-three percent self-reported high levels of PA (Category three, IPAQ-SF) and eighty-six percent met the CMOs’ PA guidelines for adults, relating to the amount of aerobic activity conducted per week.

There were no statistically significant differences observed between participants’ PA levels and whether they believed PA promotion was within the remit of their role (*p* = 0.425) or whether they had any concerns promoting it to their patients (*p* = 0.425). However, higher levels of PA were associated with knowledge of where to signpost patients for further information about PA (*Z* = − 2.7, *p* = 0.006, *r* = 0.3). There were no statistically significant associations observed between participants’ PA levels and whether they were confident initiating PA discussions (*p* = 0.206), or whether they required further training to feel confident initiating these discussions (*p* = 0.440). However, a statistically significant association was observed between participants’ own PA levels and how frequently they initiated PA discussions (*H* (5) = 14.3, *p* = 0.014, *η*^*2*^ = 0.14). Post hoc analyses revealed professionals who occasionally initiated PA discussions were more physically active, compared with those who very rarely (*Z* = − 2.9, *p* = 0.003, *r* = 0.5), or never initiated discussions (*Z* = − 2.39, *p* = 0.016, *r* = 0.4). Similarly, professionals who very frequently initiated PA discussions were more physically active, compared with those who very rarely initiated discussions (*Z* = − 2.5, *p* = 0.010, *r* = 0.5). Lastly, professionals who always initiated PA discussions were more physically active, compared with those who very rarely initiated discussions (*Z* = − 2.0,* p* = 0.049,* r* = 0.5).

There were no statistically significant associations observed between a professional’s role and whether they believed PA promotion was within the remit of their role (*p* = 0.554), or whether they had any concerns promoting PA (*p* = 0.912). There were no significant associations observed between a professional’s role and whether they were confident initiating PA discussions (*p* = 0.508), their frequency of initiating PA discussions (*p* = 0.220), or whether they required further training to feel confident initiating discussions (*p* = 0.395). However, a statistically significant association was observed between a professional’s role and their knowledge of where to signpost patients for further information about PA (*χ*^2^ (2,* N* = 71) = 6.6,* p* = 0.037, *v* = 0.3). Medical or surgical professionals were less likely to self-report knowledge of where to signpost patients for further information about PA (13%), compared with allied health (49%), or nursing (53%) professionals. Exact *p* values for inferential analyses are available in Online Resources 2a and 2b.

### Themes

Six themes were identified. Capability-related themes included ‘familiarity and use of PA guidelines and assessment tools,’ ‘lack of training received in PA promotion,’ and ‘lack of patient and professional PA discussions.’ There was one opportunity-related theme that was defined as ‘lack of physical opportunity to promote PA.’ Motivation-related themes included ‘the psychological impact of HaNC’ and ‘fear of harm.’

### Familiarity and use of PA guidelines and assessment tools (COM-B construct: psychological capability; TDF domain: knowledge)

Fifty-eight percent did not have concerns discussing PA with their patients; however, only 38% felt confident initiating these conversations. Although 52% were familiar with the CMOs’ PA guidelines, only 38% used them. In open-ended responses, some participants described the CMOs’ guidance as not appropriate for HaNC patients.

*“My understanding is very basic for the general population regarding these guidelines and may therefore not be appropriate for HaNC patients.” (HCP43; Radiography).*

Most participants (59%) were familiar with NICE PA guidelines; however, there were mixed messages as to whether any were used in clinical practice. One participant described that although they were aware there were guidelines, they did not *“know what they are” (HCP33; Dietetics)*. Most participants (58%) were familiar with the Macmillan guidelines for promoting PA for people living with and beyond cancer, but only 36% used them.

The majority were not aware of the General Practice Physical Activity Questionnaire (GPPAQ) (89%), IPAQ (95%), single-item measure (95%), Scottish PA Screening Questionnaire (Scot-PASQ) (99%), English PA Screening Questionnaire (Eng-PASQ) (95%), or device-based methods (52%), for obtaining PA data. Of those who were aware of the GPPAQ (5%), IPAQ (5%), and the Eng-PASQ (5%), 75% never used them in clinical practice. Of those who were aware of the single-item measure (5%), there was an even split as to whether the tool was sometimes used (50%) or never used (50%). Of those who were aware of the Scot-PASQ (1%), this tool was never used in clinical practice. Of those who were aware of device-based methods (48%), 51% never used them in clinical practice.

### Lack of training received in PA promotion (COM-B constructs: physical and psychological capability; TDF domains: knowledge and skills)

Only 1% had received training in the GPPAQ, the PA Clinical Champions Programme, the in-practice training session on PA, and the Clinical Commissioning Group (CCG) training session on PA. Only 2% had received training in the use of PA assessment tools, only 17% had received training in brief interventions and only 19% had received training in motivational interviewing. No participants had received training in the Royal College of General Practitioners (RCGP) accredited Continuing Medical Education (CME) module on PA, or the British Medical Journal (BMJ) PA Module. Sixty-two percent did not understand how to use PA assessment tools in their day-to-day practice. Open-ended responses indicated the use of assessment tools to examine patients’ PA levels were not useful.

*“If [I’m] honest, patients are fed up of the medical model and want to come and enjoy rehabilitation, not be assessed on it...” (HCP53; Physiotherapy).*

### Lack of patient and professional PA discussions (COM-B constructs: physical and psychological capability; TDF domains: knowledge and skills)

Only 39% reported they discussed PA with their patients in practice. In open-ended responses, participants described not knowing how to initiate these discussions and did not feel qualified enough to know what to advise patients.

*“I feel under qualified to do so! I have not had any training in this area.” (HCP19; Speech and Language Therapy).*

One participant revealed they only had these conversations when they were initiated by patients. This is particularly concerning as most participants reported these conversations were very rarely (31%), rarely (23%), or never (15%) initiated by patients.

*“I discuss this, but rather than promoting, I would wait for patients to ask me my opinion…” (HCP51; Dietetics).*

Another participant described that as patients can encounter debilitating side-effects during their treatment, discussions around PA at this time-point seem *‘misplaced*.*’*

*“During radiotherapy or [chemotherapy], they are often so unwell that this is the last thing we discuss… the patients feel as though they are just trying to survive, and discussing exercise seems misplaced.” (HCP19; Speech and Language Therapy).*

Seventy-six percent reported requiring training to feel confident initiating PA discussions, with training on how to elicit behavior change, and information on what the benefits are for HaNC, described as particularly useful.

*“Awareness and education about how to assess readiness for change, and physical baseline on which to base advice.” (HCP18; Speech and Language Therapy).*

*“…Information on what the benefits of doing this for the patient are. Patients are likely to ask why they should bother exercising when they are so unwell...” (HCP47; Radiography).*

### Lack of physical opportunity to promote PA (COM-B constructs: physical and social opportunity; TDF domains: environmental context and resources and social influences)

Seventy-seven percent reported that PA should be discussed throughout a patient’s treatment pathway, however *“it gets crowded out by more urgent issues…” (HCP17; General Practice)*.

Sixty-eight percent were unfamiliar with any exercise referral schemes available for HaNC patients and *“referrals into [an] exercise cancer specialist”* was *“very postcode dependent” (HCP1; Physiotherapy)*.

One participant described a lack of service delivery support for PA promotion from their managerial team as a result of the COVID-19 pandemic.

*“COVID-19 has limited me being able to progress with my intervention to incorporate exercise into physical and mental recovery due to… limited time from therapy staff to help with the assessment and delivery or exercise.” (HCP45; Nursing).*

### The psychological impact of HaNC (COM-B constructs: automatic and reflective motivation; TDF domains: beliefs about consequences and emotion)

As a patient’s appearance can change drastically post- treatment, patients may feel self-conscious and less likely to engage in PA, especially if it involved going into public spaces.

*“I feel a barrier would be the disfigurement of the patient, as our HaNC patients have major reconstruction and may feel embarrassed to maybe attend a gym or to even go out on a walk.” (HCP5; Nursing).*

One participant stated that many HaNC patients can become depressed as a result of their cancer. Explaining the benefits of being physically active, may help to improve patients’ psychological well-being.

*“Lots of our patients become depressed and we need to do more to avoid this in my opinion...” (HCP5; Nursing).*

### Fear of harm (COM-B constructs: automatic and reflective motivation; TDF domains: social/professional role and identity, beliefs about consequences and reinforcement)

Seventy-five percent reported it was within the remit of their role to discuss PA with their patients. However, some described being fearful of causing harm by encouraging their patients to be physically active.

*“I would be wary about encouraging too much, as we have had some patients burn too many calories by continuing their pre-treatment exercise regime which impacts on their weight.” (HCP40; Nursing).*

Moreover, participants highlighted that patients might be fearful that being physically active was not safe.

*“Fear of causing something to go wrong.” (HCP59; General Practice).*

Participants highlighted that patients needed to be encouraged that it was safe and beneficial for them to be physically active throughout their treatment pathway.

*“Educate patients that it is safe or guide them to other more appropriate forms of exercise.” (HCP48; Speech and Language Therapy).*

## Discussion

### Summary of main findings

*Reflective motivation, psychological capability*, and the TDF domains *social/professional role and identity* and *knowledge* were key barriers to PA promotion. Despite the majority of professionals considering PA promotion as part of their role, PA was not routinely discussed with HaNC patients. Similarly, although the majority were familiar with PA guidelines for promoting PA, the majority did not use them in clinical practice. Exploratory quantitative analyses found that professionals with higher levels of PA were more knowledgeable of where to signpost patients for further information about PA. Professionals who discussed PA more frequently with their patients, were more physically active in comparison with those who very rarely, or never initiated these discussions. Medical professionals were less likely to self-report knowledge of where to signpost patients for further information about PA, compared with allied health, or nursing professionals. Professionals identified the need for further training on the benefits of PA for HaNC patients and how to encourage health-related behavior change.

### Comparison with previous literature

Although PA promotion should be a standard part of cancer care [14], the current study identified that only 38% felt confident initiating these conversations and only 39% discussed PA in practice. However, this differs from previous research conducted in the USA that reported 70% of healthcare professionals often or routinely promoted PA to their patients with cancer [44]. However, this previous study was conducted with professionals predominately working with breast, prostate, colorectal, and lung cancer patients. Medical professionals were less likely to self-report knowledge of where to signpost patients for further information about PA, compared with allied health or nursing professionals. This contradicts the findings from a previous survey conducted in England that reported that allied health professionals reported low confidence in providing PA advice to their patients with cancer [26]. Exploratory analyses revealed significant effects for professionals’ own PA levels and the extent to which they understood how to use PA assessment tools in day-to-day practice, their frequency of PA discussions, their knowledge of where to signpost patients for further information, and whether they used PA guidelines or exercise referral schemes in clinical practice. These findings are consistent with previous research indicating that healthcare professionals who met PA guidelines themselves, were more likely to provide PA advice, discuss, and refer cancer patients, including those with HaNC, to a PA programme or specialist [18]. The current study identified that as HaNC patients can experience debilitating treatment-related side effects, professionals thought PA discussions were misplaced during consultations. However, as PA can decrease fatigue, improve functional well-being and quality of life for HaNC patients [7–9], reassuring patients that it is safe and beneficial to be physically active, is important for improving health-related outcomes.

## Strengths and limitations

This was the first cross-sectional study to use the COM-B and TDF to explore healthcare professionals’ PA promotion for HaNC. The use of quantitative and qualitative data allowed for detailed responses to be collected and provided context to the information provided. As the present sample size was relatively small for group comparisons, the inferential analyses were exploratory and hypothesis building and should not be interpreted as conclusive. Data were collected from regional organizations across the North West of England and North Wales and may not be generalizable to healthcare systems outside of the UK.

## Implications for practice and future research

PA promotion was not routine practice for professionals involved in the care of HaNC patients in the UK NHS organizations surveyed. Despite professionals self-reporting they were aware of PA guidelines, the guidelines were not used in clinical practice. Time constraints during consultations may prevent professionals from promoting PA. Clinical Exercise Physiologists (CEP) [45] are tertiary-qualified healthcare professionals specializing in the prescription of PA interventions [45] and they may be able to facilitate physical activity promotion. However, to ensure all professionals feel confident promoting PA, it is essential to provide information and training, incorporating behavior change techniques that focus on *psychological capability* and the TDF domain *knowledge*.

## Conclusion

Healthcare professionals are influential in enabling patients to adopt health-related behavior change; however, they can lack the knowledge and confidence required to promote PA. Subsequently, low rates of PA participation in HaNC patients may reflect lack of promotion, knowledge, and support. CEP’s can facilitate physical activity promotion; however, training should be provided to all professionals, with a focus on behavior change techniques. Future research should explore how PA behavior change techniques can be implemented into clinical practice, to improve acute and long-term outcomes for HaNC patients.

## Supplementary Information

Below is the link to the electronic supplementary material.

Supplementary file1 (DOCX 72 KB)

## Data Availability

The data to support the findings of this research are available upon reasonable request.
