# Supplementary material for: Healthcare professionals’ promotion of physical activity to people living with and beyond head and neck cancer: a cross-sectional survey
Source: Support Care Cancer. 2024 Dec 3;32(12):848. doi: 10.1007/s00520-024-09027-8 (PMC11611970; doi:10.1007/s00520-024-09027-8)
Supplement: Supplementary file 1 — Supplementary file1 (DOCX 72 KB) [file 520_2024_9027_MOESM1_ESM.docx]

**Online Resource 1.** Pilot Survey

| **Survey Item** |
| --- |
| 1. What gender do you identify as? |
| Female |
| Male |
| Transgender |
| Non-binary |
| Prefer not to say |
| 1. What is your sexuality? |
| Heterosexual |
| Homosexual |
| Bisexual |
| Other |
| Prefer not to say |
| 1. What is your ethnicity? |
| White – English/ Welsh/ Irish/ Gypsy or Irish Traveller |
| Mixed/Multiple Ethnic Groups– White and Black Caribbean |
| Mixed/Multiple Ethnic Groups – White and Black African |
| Mixed/Multiple Ethnic Groups – White and Asian |
| Asian/Asian British – Indian |
| Asian/Asian British – Pakistani |
| Asian/Asian British – Bangladeshi |
| Asian/Asian British – Chinese |
| Black/African/Caribbean/Black British – African |
| Black/African/Caribbean/Black; British – Caribbean |
| Other Ethnic Group – Arab |
| Other Ethnic Group – Any Other Ethnic Group |
| Prefer not to say |
| 1. What is your age? |
| Open-ended response |
| Prefer not to say |
| 1. Approximately how long have you worked as a healthcare professional? |
| Open-ended response |
| Prefer not to say |
| 1. Approximately how long have you worked with head and neck cancer patients? |
| Open-ended response |
| Prefer not to say |
| 1. What is your current job role? |
| Open-ended response |
| Prefer not to say |
| 1. Where is your location of practice? |
| Open-ended response |
| Prefer not to say |
| 1. Approximately how many head and neck patients do you see a month? |
| Open-ended response |
| Prefer not to say |
| 1. What is the name of the organisation you work in? |
| Open-ended response |
| Prefer not to say |
| 1. Do you think it is important to discuss physical activity with your head and neck cancer patients? |
| Yes |
| No |
| Prefer not to say |
| Optional open-text box for elaboration |
| 1. For patients with which of the following health conditions would you discuss and recommend physical activity to? *(Yes/no response option for each condition)* |
| Overweight (BMI >25 and <30) |
| Type 2 diabetes |
| Mild or moderate depression |
| Weight maintenance following weight loss |
| Hypertension |
| Osteoarthritis and other joint pain |
| Distress |
| Stress or anxiety |
| Chronic fatigue |
| Reduced bone density/risk of bone fracture |
| Muscular/joint pain |
| Hyperlipidaemia |
| Sleep disorder |
| Post-stroke |
| Asthma |
| Post-natal |
| Continuation during pregnancy |
| Before pregnancy (pre-conception advice) |
| Dementia or other cognitive decline |
| Irritable bowel syndrome |
| Substance misuse withdrawal |
| Initiation during pregnancy |
| With diagnosed cancer |
| Other |
| Prefer not to say |
| Optional open-text box for elaboration |
| 1. I never discuss physical activity with my patients |
| True |
| False |
| Prefer not to say |
| Optional open-text box for elaboration |
| 1. I rarely discuss physical activity with my patients |
| True |
| False |
| Prefer not to say |
| Optional open-text box for elaboration |
| 1. I discuss physical activity with my patients |
| True |
| False |
| Prefer not to say |
| Optional open-text box for elaboration |
| 1. To your knowledge, do you think physical activity is discussed with head and neck cancer patients in your speciality? *Please elaborate on your answers where possible* |
| Yes |
| No |
| Prefer not to say |
| Optional open-text box for elaboration |
| 1. Who do you think has, or should have these discussions with patients? |
| Open-ended |
| Prefer not to say |
| 1. Do you think it is within the remit of your role to discuss physical activity with head and neck cancer patients? |
| Yes |
| No |
| Prefer not to say |
| Optional open-text box for elaboration |
| 1. Do you have any concerns discussing physical activity with head and neck cancer patients, or signposting patients to existing physical activity provision in the area? |
| Yes |
| No |
| Prefer not to say |
| Optional open-text box for elaboration |
| 1. When do you think would be an appropriate time to discuss physical activity with head and neck cancer patients?* |
| At one time-point |
| At all time-points (at diagnosis, pre-treatment, during-treatment, and post-treatment) |
| Other |
| Prefer not to say |
| Optional open-text box for elaboration |
| 1. What in your opinion do you think are the barriers that head and neck cancer patients may face when being physically active? |
| Open-ended response |
| Prefer not to say |
| 1. In your opinion, what do you think could be done to overcome these barriers? |
| Open-ended response |
| Prefer not to say |
| 1. A) Are you familiar with the following guidelines or exercise referral schemes? 2. If familiar, do you use the guideline(s) or exercise referral schemes?** |
| 1. Chief Medical Officers’ (CMO) Physical Activity Guidelines? |
| Not at all familiar |
| Slightly familiar |
| Somewhat familiar |
| Moderately familiar |
| Extremely familiar |
| Prefer not to say |
| Optional open-text box for elaboration |
| 1. I use these guidelines** |
| Strongly disagree |
| Disagree |
| Neither agree nor disagree |
| Agree |
| Prefer not to say |
| Optional open-text box for elaboration |
| 1. Are you familiar with any of the National Institute for Heath and Care Excellence (NICE) guidelines? |
| Not at all familiar |
| Slightly familiar |
| Somewhat familiar |
| Moderately familiar |
| Extremely familiar |
| Prefer not to say |
| Optional open-text box for elaboration |
| 1. Which guideline(s) are you aware of AND do you use them?** |
| Open-ended response |
| Prefer not to say |
| 1. Are you familiar with the Macmillan guidelines for promoting physical activity to people living with and beyond cancer? |
| Not at all familiar |
| Slightly familiar |
| Somewhat familiar |
| Moderately familiar |
| Extremely familiar |
| Prefer not to say |
| Optional open-text box for elaboration |
| 1. I use these guidelines** |
| Strongly disagree |
| Disagree |
| Neither agree nor disagree |
| Agree |
| Strongly agree |
| Prefer not to say |
| Optional open-text box for elaboration |
| 1. Are you familiar with any exercise referral schemes available for people living with and beyond head and neck cancer? |
| Not at all familiar |
| Slightly familiar |
| Somewhat familiar |
| Moderately familiar |
| Extremely familiar |
| Prefer not to say |
| Optional open-text box for elaboration |
| 1. Which guideline(s) are you aware of AND do you use them?** |
| Open-ended response |
| Prefer not to say |
| 1. A) Which, if any, of the following physical activity assessment tools are you aware of to help assess patients’ physical activity levels?   B) If aware, do you use the tool(s)?** |
| 1. General Practice Physical Activity Questionnaire (GPPAQ) |
| Yes |
| No |
| Prefer not to say |
| Optional open-text box for elaboration |
| 1. If aware, do you use it?** |
| Frequently |
| Sometimes |
| Never |
| Prefer not to say |
| Optional open-text box for elaboration |
| 1. International Physical Activity Questionnaire (IPAQ) |
| Yes |
| No |
| Prefer not to say |
| Optional open-text box for elaboration |
| 1. If aware, do you use it?** |
| Frequently |
| Sometimes |
| Never |
| Prefer not to say |
| Optional open-text box for elaboration |
| 1. Single-item measure for physical activity |
| Yes |
| No |
| Prefer not to say |
| Optional open-text box for elaboration |
| 1. If aware, do you use it?** |
| Frequently |
| Sometimes |
| Never |
| Prefer not to say |
| Optional open-text box for elaboration |
| 1. Scottish Physical Activity Screening Questionnaire (Scot-PASQ) |
| Yes |
| No |
| Prefer not to say |
| Optional open-text box for elaboration |
| 1. If aware, do you use it?** |
| Frequently |
| Sometimes |
| Never |
| Prefer not to say |
| Optional open-text box for elaboration |
| 1. English Physical Activity Screening Questionnaire (Eng-PASQ) |
| Yes |
| No |
| Prefer not to say |
| Optional open-text box for elaboration |
| 1. If aware, do you use it?** |
| Frequently |
| Sometimes |
| Never |
| Prefer not to say |
| Optional open-text box for elaboration |
| 1. Device-based methods for obtaining physical activity data (e.g., Fitbit, pedometer) |
| Yes |
| No |
| Prefer not to say |
| Optional open-text box for elaboration |
| 1. If aware, do you use it?** |
| Frequently |
| Sometimes |
| Never |
| Prefer not to say |
| Optional open-text box for elaboration |
| 1. I understand how to use these assessment tools in day-to-day practice |
| Strongly disagree |
| Disagree |
| Neither agree nor disagree |
| Agree |
| Strongly agree |
| Prefer not to say |
| Optional open-text box for elaboration |
| 1. Do you know where to signpost head and neck cancer patients for further information about physical activity? |
| Yes |
| No |
| Prefer not to say |
| Optional open-text box for elaboration |
| 1. I am confident initiating physical activity discussions with head and neck cancer patients |
| Strongly disagree |
| Disagree |
| Neither agree nor disagree |
| Agree |
| Strongly agree |
| Prefer not to say |
| Optional open-text box for elaboration |
| 1. I feel that I need further training in order to feel confident initiating discussions about physical activity with my head and neck cancer patients |
| Strongly disagree |
| Disagree |
| Neither agree nor disagree |
| Agree |
| Strongly agree |
| Prefer not to say |
| Optional open-text box for elaboration |
| 1. What training do you think you or your clinical practice would benefit from? |
| Open-ended response |
| Prefer not to say |
| 1. A) Which, if any, of the following training sessions have you undertaken with respect to encouraging physical activity?   B) If you have received training, do you feel more confident?** |
| 1. Using General Practice Physical Activity Questionnaire (GPPAQ) in-practice |
| Yes |
| No |
| Prefer not to say |
| Optional open-text box for elaboration |
| 1. If you have received training, do you feel more confident?** |
| Yes |
| Somewhat |
| No |
| Prefer not to say |
| Optional open-text box for elaboration |
| 1. Delivering brief interventions to encourage patient’s physical activity |
| Yes |
| No |
| Prefer not to say |
| Optional open-text box for elaboration |
| 1. If you have received training, do you feel more confident?** |
| Yes |
| Somewhat |
| Prefer not to say |
| Optional open-text box for elaboration |
| 1. Motivational interviewing |
| Yes |
| No |
| Prefer not to say |
| Optional open-text box for elaboration |
| 1. If you have received training, do you feel more confident?** |
| Yes |
| Somewhat |
| No |
| Prefer not to say |
| Optional open-text box for elaboration |
| 1. Use of physical activity assessment tools |
| Yes |
| No |
| Prefer not to say |
| Optional open-text box for elaboration |
| 1. If you have received training, do you feel more confident?** |
| Yes |
| Somewhat |
| No |
| Prefer not to say |
| Optional open-text box for elaboration |
| 1. Clinical Commissioning Group (CCG) training session on physical activity |
| Yes |
| No |
| Prefer not to say |
| Optional open-text box for elaboration |
| 1. If you have received training, do you feel more confident?** |
| Yes |
| Somewhat |
| No |
| Prefer not to say |
| Optional open-text box for elaboration |
| 1. In-practice training session on physical activity |
| Yes |
| No |
| Prefer not to say |
| Optional open-text box for elaboration |
| 1. If you have received training, do you feel more confident?** |
| Yes |
| Somewhat |
| No |
| Prefer not to say |
| Optional open-text box for elaboration |
| 1. Royal College of General Practitioners (RCGP) accredited Continuing Medical Education (CME) module on physical activity |
| Yes |
| No |
| Prefer not to say |
| Optional open-text box for elaboration |
| 1. If you have received training, do you feel more confident?** |
| Yes |
| Somewhat |
| No |
| Prefer not to say |
| Optional open-text box for elaboration |
| 1. British Medical Journal (BMJ) Physical Activity Module |
| Yes |
| No |
| Prefer not to say |
| Optional open-text box for elaboration |
| 1. If you have received training, do you feel more confident?** |
| Yes |
| Somewhat |
| No |
| Prefer not to say |
| Optional open-text box for elaboration |
| 1. Physical Activity Clinical Champions Programme |
| Yes |
| No |
| Prefer not to say |
| Optional open-text box for elaboration |
| 1. If you have received training, do you feel more confident?** |
| Yes |
| Somewhat |
| No |
| Prefer not to say |
| Optional open-text box for elaboration |
| 1. The discussions I have about physical activity with my head and neck cancer patients have changed since COVID-19 |
| Strongly disagree |
| Disagree |
| Neither agree nor disagree |
| Agree |
| Strongly agree |
| Prefer not to say |
| Optional open-text box for elaboration |
| 1. Can you briefly summarise what has changed?** |
| Open-ended response |
| Prefer not to say |
| 1. How frequently do your head and neck cancer patients initiate discussions about physical activity with you? |
| Never |
| Very rarely |
| Rarely |
| Occasionally |
| Very frequently |
| Prefer not to say |
| Optional open-text box for elaboration |
| 1. Approximately how frequently have you discussed physical activity with your head and neck cancer patients in the last month? |
| Never |
| Very rarely |
| Rarely |
| Occasionally |
| Very frequently |
| Always |
| Prefer not to say |
| Optional open-text box for elaboration |
| IPAQ-SF Questionnaire |
| Seven questionnaire items |

** Multiple response options could be selected*

*** Question contingent on the previous response*

**Online Resource 2a.** Exact *p* Values for All Inferential Analyses

| **Mann-Whitney** | | | | | | | | |
| --- | --- | --- | --- | --- | --- | --- | --- | --- |
| **Independent Variable** | **Significance Level** | **Dependent Variables** | | | | | | |
|  | | (Q11) Remit of role to discuss physical activity | (Q12) Concerns discussing physical activity | (Q21) Knowledge of where to signpost patients for information about physical activity |  | | | |
| **Met-min/week** | **Significance (two-sided)** | 0.425 | 0.425 | 0.006****** |  |  | |  |
| **Kruskal-Wallis** | | | | | | | | |
| **Independent Variable** | **Significance Level** | **Dependent Variables** | | | | | | |
|  | | (Q22) Confidence initiating physical activity discussions | (Q29) Frequency of  physical activity discussions | (Q23) Further training required to feel confident initiating physical activity discussions |  | | | |
| **Met-min/week** | **Significance (two-sided)** | 0.206 | 0.014****** | 0.440 |  |  | |  |
| **Chi-Square** | | | | | | | | |
| **Independent Variable** | **Significance Level** | **Dependent Variables** | | | | | | |
|  | | (Q11) Remit of role to discuss physical activity | (Q12) Concerns discussing physical activity | (Q21) Knowledge of where to signpost patients for information about physical activity | (Q22) Confidence initiating physical activity discussions | (Q29) Frequency of  physical activity discussions | (Q23) Further training required to feel confident initiating physical activity discussions | |
| **Healthcare professional role** | **Significance (two-sided)** | 0.554 | 0.912 | 0.037* | 0.508 | 0.220 | 0.395 | |

**Significance level p < 0.05*

***Significance level p ≤ 0.01*

**Online Resource 2b**. Exact *p* Values for Post-Hoc Analyses

| **Mann-Whitney** | | | | | | |
| --- | --- | --- | --- | --- | --- | --- |
| **Independent Variable** | **Significance Level** | **Dependent Variable**  ***(Frequency of medical or allied health professional initiated physical activity discussions)*** | | | | |
|  | | (Q29) Never or very rarely initiated physical activity discussions | (Q29) Never or rarely initiated physical activity discussions | (Q29) Never or occasionally initiated physical activity discussions | (Q29) Never or very frequently initiated physical activity discussions | (Q29) Never or always initiated physical activity discussions |
| **Met-min/week** | **Significance (two-sided)** | 0.621 | 0.781 | 0.016* | 0.053 | 0.127 |
|  | | (Q29) Very rarely or rarely initiated physical activity discussions | (Q29) Very rarely or occasionally initiated physical activity discussions | (Q29) Very rarely or very frequently initiated physical activity discussions | (Q29) Very rarely or always initiated physical activity discussions | (Q29) Rarely or occasionally initiated physical activity discussions |
| **Met-min/week** | **Significance (two-sided)** | 0.456 | 0.003** | 0.010** | 0.049* | 0.101 |
|  | | (Q29) Rarely or very frequently initiated physical activity discussions | (Q29) Rarely or always initiated physical activity discussions | (Q29) Occasionally or very frequently initiated physical activity discussions | (Q29) Occasionally or always initiated physical activity discussions | (Q29) Very frequently or always initiated physical activity discussions |
| **Met-min/week** | **Significance (two-sided)** | 0.169 | 0.212 | 0.962 | 0.907 | 0.604 |

**Significance level p < 0.05*

***Significance level p ≤ 0.01*
